# Exploring the Interpretive Clarity of the TCCNI-RePract and Identifying Conceptual Barriers Encountered by Japanese Psychiatric Nurses: A Concurrent Mixed-Methods Study

**DOI:** 10.3390/nursrep16030077

**Published:** 2026-02-24

**Authors:** Yoshiyuki Takashima, Gil Platon Soriano, Allan Paulo Blaquera, Hirokazu Ito, Yuko Yasuhara, Kyoko Osaka, Tetsuya Tanioka

**Affiliations:** 1 Faculty of Nursing, School of Medicine, Nara Medical University, Nara 634-0813, Japan; 2 Department of Nursing, College of Allied Health, National University Philippines, Manila 1008, Philippines; gil.p.soriano@gmail.com; 3 School of Nursing and Allied Health Sciences, St. Paul University, Tuguegarao City 3500, Philippines; ablaquera@spup.edu.ph; 4 Graduate School of Biomedical Sciences, Tokushima University, Tokushima 770-8509, Japan; h.itoh@tokushima-u.ac.jp (H.I.); yasuhara@tokushima-u.ac.jp (Y.Y.); tanioka.tetsuya@tokushima-u.ac.jp (T.T.); 5 Department of Nursing, Nursing Course of Kochi Medical School, Kochi University, Kochi 783-8505, Japan; osaka@kochi-u.ac.jp

**Keywords:** psychiatric nursing, technological competency as caring in nursing, thematic analysis in-service education

## Abstract

**Background/Objectives**: Integrating technology with caring is essential in modern healthcare, yet the clinical applicability of nursing theories remains underexplored. Locsin’s Technological Competency as Caring in Nursing (TCCN) theory emphasizes the competent use of technology to address patients holistically, rather than focusing solely on health concerns. Here, we explored the interpretive clarity of the TCCN Instrument–Revised for Practice (TCCNI-RePract) items and identified the conceptual barriers encountered by psychiatric nurses when engaging with its theoretical constructs. **Methods**: This concurrent mixed-methods study surveyed 291 psychiatric nurses across five large hospitals in the Kansai region of Japan. Quantitative data on the TCCNI-RePract perception dimension were examined using descriptive statistics and normality testing. Qualitative open-ended responses were analyzed using reflexive thematic analysis. To ensure rigor and integration, a joint display was utilized to bridge both data strands. **Results**: Quantitative findings indicated that nurses strongly endorsed core values of caring (high agreement) but perceived theoretical constructs (wholeness and technological knowing) as significantly more difficult to interpret than concrete, behavior-oriented items. Qualitative analysis revealed four major themes: (1) fragmented understanding of “technology and caring,” (2) struggles with abstract and philosophical language, (3) moral and emotional tensions in caring relationships, and (4) contextual barriers to integrating caring and technology. We found a “semantic gap,” where the professional endorsement of caring values was not automatically translated into the mastery of theoretical lexicon. **Conclusions**: While psychiatric nurses identify with the moral core of TCCN, a substantial gap exists between abstract theory and clinical practice. For effectiveness, middle-range theories require “clinical translation” that resonates with the moral, emotional, and organizational realities of psychiatric settings.

## 1. Introduction

Advances in imaging (e.g., computed tomography, magnetic resonance imaging, optical topography) [1,2], medical testing devices, mobile applications [3], wearable technology, and remote sensors have increasingly shaped psychiatric care [4,5]. These technological advancements have positively affected human health by enabling nurses to work more effectively and safely [6]. However, as technology becomes more pervasive, a critical challenge emerges: the shifting of treatment focus from the patient as a whole individual toward the technical operation of devices. This can result in nurses’ failure to understand the patient as a person [7,8] and may widen the gap in interpersonal contact [8]. Addressing this tension is not merely a philosophical exercise but rather a practical urgency in modern psychiatry to prevent the dehumanization of care.

The Technological Competency as Caring in Nursing (TCCN) theory developed by Locsin proposes a contemporary approach that integrates technology with nursing care [6]. When used competently, technology in nursing can facilitate “technological knowing” [7], which involves the use of technology to understand patients as whole beings whose hopes, dreams, and aspirations are important for them to live their lives to the fullest [8]. Nevertheless, when the technical skills of nurses lack a theoretical foundation, the care recipients (patients and their families) are more likely to be treated as objects [6]. Therefore, examining how nurses perceive and interpret these theoretical constructs is essential for sustainable integration of technology in nursing practice.

Japan provides a unique and crucial context for this exploration. Befitting of a society at the forefront of healthcare technology integration, Japanese psychiatric hospitals frequently employ surveillance systems and monitoring devices, particularly during seclusion and restraint procedures. Nurses in these settings often encounter morally distressing situations [9,10], and ethical dilemmas [11]. While patients must be considered irreplaceable in person-centered practice, the specific cognitive and linguistic barriers that prevent nurses from articulating their practice through a theoretical lens remain poorly understood. We previously developed a psychiatry-specific instrument that assesses the practice of the TCCN theory [6]. However, initial observations suggested a potential “semantic gap” between the theory’s abstract lexicon and the pragmatic clinical reality. Clarifying how psychiatric nurses navigate these constructs is crucial for improving the quality of care. Rather than assuming the theory’s immediate applicability, arising conceptual difficulties should be identified.

While previous studies on TCCN have primarily focused on philosophical discourse and theoretical validation, empirical evidence regarding how clinical nurses perceive and interpret these complex constructs remains scarce. This study is the first to address this critical gap by employing a mixed-methods design to specifically identify the elements of TCCN perceived as “difficult to understand” by frontline psychiatric nurses. By pinpointing these conceptual hurdles, this research moves beyond abstract advocacy of the theory, providing actionable insights for the “clinical translation” of TCCN into pragmatic nursing practice.

Thus, we aimed to explore the interpretive clarity of the TCCN Instrument–Revised for Practice (TCCNI-RePract) items and identify the conceptual barriers encountered by psychiatric nurses when engaging with its theoretical constructs.

## 2. Materials and Methods

### 2.1. Research Design

This study adopted a concurrent mixed-methods approach, in which quantified item-level perceived difficulty data were obtained from the perception dimension of the 25-item TCCNI-RePract and a thematic analysis was conducted on the participants’ open-ended comments.

To clarify the scope, this study focused on “perceived interpretive difficulty” rather than psychometric item difficulty as defined in item response theory (IRT) or formal instrument validation. Perceived interpretive difficulty refers to the cognitive, linguistic, and contextual barriers encountered by psychiatric nurses when interpreting theoretical constructs in a clinical setting. This approach was chosen to explore the “semantic gap” between the abstract nursing theory and everyday psychiatric practice, which requires a nuanced analysis of the subjective perspectives of participants through mixed-methods integration.

### 2.2. Participants, Settings, and Criteria

Japan provides a critical setting for this study owing to its status as a global leader in healthcare technology integration, contrasted with a nursing culture that deeply values traditional, high-touch interpersonal caring. Specifically, Japanese psychiatric care increasingly relies on advanced surveillance and monitoring systems for safety management during high-risk procedures, such as seclusion and restraint. This creates a unique clinical environment where nurses must navigate the tension between technological efficiency and human-centered caring.

Psychiatric nurses across five large psychiatric hospitals (each with over 300 beds) in the Kansai region of Japan were recruited from November 2022 to April 2023. These hospitals were selected through purposive sampling, and participation was secured via direct contact with nursing administrators. To minimize selection bias, nursing administrators were requested to distribute the survey to all eligible staff, regardless of their familiarity with nursing theory.

Inclusion criteria were being a registered nurse (RN) or licensed practical nurse (LPN) currently employed in a psychiatric ward at one of the target institutions. Exclusion criteria were (1) less than one year of psychiatric nursing experience, as inexperience nurses might still be in the process of clinical orientation, and (2) submission of incomplete surveys.

A total of 310 nurses responded. Following the exclusion of 19 respondents based on the selection criteria (12 owing to insufficient experience and seven owing to incomplete responses), 291 valid responses were included in the final analysis.

### 2.3. Instrumentation

The survey comprised two parts, namely, personal characteristics and the TCCNI-RePract questionnaire. The participants were asked to provide information on their personal attributes, including their sex, age, years of experience as a nurse and psychiatric nurse, nursing qualifications, and current ward where they were working.

The TCCNI-RePract evaluates whether nurses perceive and practice the TCCN theory in clinical practice arenas. This instrument consists of 25 items for the perception dimension, and its reliability and validity have been tested (Cronbach’s α = 0.94 for the total scale). Satisfactory construct validity has also been confirmed through confirmatory factor analysis (χ^2^/df ratio = 2.13, comparative fit index = 0.93, root mean square error of approximation = 0.05) [12]. The perception dimension is measured using a 7-point Likert scale, with values ranging from 1 (strongly disagree) to 7 (strongly agree) [12]. In the present study, the participants completed the perception dimension of the TCCNI-RePract to assess their understanding of this instrument. A free-text section with the prompt “*Please describe any aspects of the questionnaire items that you find difficult or hard to understand*” was included to further explore the reasons why certain items were perceived as difficult. Permission to use the TCCNI-RePract instrument was obtained from the original authors before data collection.

### 2.4. Data Collection

Nursing administrators facilitated survey distribution using both paper- and web-based methods (SurveyMonkey^®^). The participants could choose their preferred format. Each online entry was limited to one response per participant to ensure data integrity and accuracy. Additionally, the potential for selection bias owing to clustering by facility was considered.

### 2.5. Data Analysis

The demographic characteristics of the study participants are expressed as frequencies (*n*) and percentages (%) (Table 1). Quantitative analyses were performed using IBM SPSS Statistics version 29.0 (IBM Corp., Armonk, NY, USA). Missing data (<5%) were handled through listwise deletion. The median and interquartile range (IQR) for the participants’ perception of the TCCNI-RePract were calculated. The normality of continuous variables, including age, years of experience, and TCCNI-RePract scores, was assessed using the Shapiro–Wilk test. As the assumption of normality was not met for these variables, data are expressed as medians and IQRs.

TCCNI-RePract items not understood by the participants or perceived by the participants to be difficult to understand are enumerated in Table 2. The selection criterion was considered as difficult to understand by at least five participants.

This study employed a concurrent mixed-methods design, utilizing a pillar integration process to connect the quantitative and qualitative strands. To move beyond a mere parallel reporting of results, we used the identified “difficult items” from the quantitative strand to strategically focus the qualitative inquiry. Items were selected for prioritized qualitative analysis if they were reported as difficult to understand by at least five participants (n ≥ 5). This threshold was employed not as a measure of statistical significance for generalization but as a pragmatic exploratory filter to ensure that the subsequent thematic analysis focused on recurring, shared interpretive patterns rather than idiosyncratic misunderstandings. A sensitivity check (testing the thresholds of n ≥ 3 and n ≥ 10) confirmed that the resulting themes remained stable, ensuring the robustness of the findings.

The six-phase method outlined by Braun and Clarke was employed to analyze open-ended survey responses using reflexive thematic analysis [13,14]. Thematic analysis is a flexible technique used to identify and understand meaningful patterns in qualitative data. Particularly, reflexive thematic analysis highlights the active role of researchers in co-constructing meaning from the data, recognizing that meaning is not objectively discovered but rather developed through interaction [13]. This method is particularly useful in determining how psychiatric nurses interpret theoretical concepts pertaining to technology and care.

In this study, preliminary codes were generated inductively, and themes were refined iteratively to ensure coherence and consistency with the research topic. Finally, through an “interrogative integration” process, the qualitative insights were used to scrutinize the conceptual boundaries of the TCCNI-RePract, identifying the reasons underlying the quantitative findings.

### 2.6. Trustworthiness, Transparency, and Reproducibility

Specific steps were implemented to ensure the trustworthiness of qualitative findings. Credibility was enhanced through prolonged engagement with the data by two independent researchers (Y.T. and G.S.), who repeatedly read and coded the responses. Peer debriefing was regularly conducted, in which the two coders discussed any disagreements and refined themes until a consensus was reached, ensuring that the themes accurately represented the participants’ perspectives (internal agreement). Furthermore, confirmability was addressed by maintaining an audit trail of the coding process, theme development, and memos to demonstrate that the conclusions were derived directly from the data (data-to-conclusion link). Direct participant quotes were also used in presenting the findings to support the developed themes. Finally, rich and detailed descriptions of the study context, participants (Table 1), and data analysis process were provided to promote transferability.

Reflexivity: The researchers acknowledge their dual roles as proponents of the TCCN theory and investigators of its clinical clarity. To ensure analytical independence and mitigate confirmation bias, the team adopted a critical-realist stance. We proceeded with the conviction that for TCCN—a middle-range theory—to be genuinely valuable, it must withstand the scrutiny of clinical practitioners. Therefore, our analysis intentionally focused on identifying failures in conceptual translation. To safeguard credibility, (1) primary coding was cross-checked by a researcher (G.S.) not involved in the original theory development, and (2) during peer debriefing, we actively sought “discrepant cases” in which nurse responses suggested that TCCN constructs were not merely “difficult” but potentially misaligned with the pragmatic realities of psychiatric nursing.

All analyses followed the Strengthening the Reporting of Observational Studies in Epidemiology (STROBE) guidelines [15] for cross-sectional studies to ensure transparency and reproducibility (Table S1), and the reporting of mixed-methods components adhered to the Good Reporting of a Mixed Methods Study (GRAMMS) guidelines (Table S2) [16].

## 3. Results

### 3.1. Quantitative Findings

#### 3.1.1. Demographic Characteristics of the Participants

The demographic characteristics of the participants are summarized in Table 1. A self-administered questionnaire was distributed to 310 participants, among whom 291 responded and were included in the analysis.

#### 3.1.2. Items in the Perception Dimension of the TCCNI-RePract That Were Rated as Difficult to Understand by Participants

The items perceived by psychiatric nurses to be difficult to understand are listed in Table 2. Out of 25 items of the perception dimension of the TCCNI-RePract, 21 were identified as difficult to understand by at least one participant, and 12 were rated as difficult to understand by five or more participants. Items Q6, Q7, Q9, Q15, and Q16 were not regarded as difficult to understand. The median scores ranged from 5.00 to 7.00, indicating moderate to high agreement with the instrument’s statements despite the reported interpretive difficulty.

The single most cited item was Q22 (“Nursing as caring is the involvement of nurses with patients and their families in ways that allow them to grow together in the shared nursing situation”; n = 19), followed by Q20 (“Nurses’ competence includes the use of healthcare technologies from the perspective of caring in nursing”; n = 15) and Q2 (“Nurses are professionals who express caring utilizing competency with technology”; n = 8). Concept-integration prompts also appeared in the difficult set, including Q4 (“Nurses must provide nursing care through the harmonious relationship between technological knowing and caring”; n = 6) and Q10 (“Nurses have to use technology in order to know patients as persons who are complete and to maintain honest relationships with them”; n = 7). Items invoking wholeness (Q21; n = 7) and epistemic use of technology to know patients (Q23; n = 6) or their families (Q24; n = 5) were likewise problematic. In contrast, operational/behavioral items such as Q6 (co-planning care with patients; n = 0), Q7 (needing health data; n = 0), Q9 (providing care with awareness of one’s competence; n = 0), Q15 (providing timely care according to patients’ condition; n = 0), and Q16 (devotion to needs, hopes, wishes, and dreams; n = 0) were not flagged.

### 3.2. Qualitative Findings

Four broad themes emerged from the open-ended survey responses (*N* = 68), which explained how psychiatric nurses viewed, understood, and applied the theoretical concepts of TCCN.

#### 3.2.1. Theme 1: Fragmented Understanding of “Technology and Caring”

A fragmented understanding of “Technology and Caring” was identified. Of the 68 nurses who responded, 42 (61.8%) were uncertain about the definition of “technology” and how it relates to providing care in the TCCNI-RePract context.

Their responses indicated that technology was often narrowly perceived as a collection of instruments, gadgets, or data systems employed in therapeutic work, rather than being associated with caring relationships. Some participants questioned whether the use of technology constituted caring, whereas others focused on information management or tangible equipment.

Some participants mentioned the following:

*“I did not understand the meaning of ‘technology’ very well. Does it mean physical data? Infusion pumps? I didn’t understand the definition.”*

*“Some words were a bit difficult, like ‘caring’ and ‘technology.’ They feel like different things.”*

These findings revealed that nurses perceived the concepts of technology and caring as distinct in their daily practice. In particular, caring was viewed as emotional and relational, whereas technology was typically associated with tasks and efficiency.

#### 3.2.2. Theme 2: Struggles with Abstract and Philosophical Language

A total of 25 nurses (36.8%) reported difficulty in comprehending theoretical or abstract terms that were challenging to apply in their daily work. Words such as “personhood,” “sincerity,” “wholeness,” and “hope” were viewed as ambiguous and subject to interpretation. Numerous nurses expressed that providing meaningful answers was difficult because the phrasing of several questions seemed disconnected from practical reality.

Some statements were as follows:

*“It was hard to answer questions involving abstract concepts or those that could change depending on personal interpretation.”*

*“I was unsure how to interpret words like compassion, sincerity, hope, and dreams.”*

These findings revealed a preference for practical action-oriented expressions of caring over academic or philosophical explanations.

#### 3.2.3. Theme 3: Moral and Emotional Tensions in Caring Relationships

A total of six nurses (9.8%) described experiences reflecting moral and emotional tensions in caring relationships. Strong emotions and moral introspection were evident in the responses. Some nurses felt uneasy when they read things that seemed to imply that they might not have fully understood their patients or ignored certain areas of care. Instead of viewing these items as theoretical cues, they perceived them as personal assessments that made them feel guilty or defensive.

Some statements were as follows:

*“In a question like No. 11, it sounded as if I was always neglecting patients.”*

*“Understanding patients’ feelings cannot be achieved in one day.”*

*“Nurses are humans too, with their own lives.”*

These findings suggested that caring was viewed as both a moral and personal endeavor, in addition to being a professional obligation.

#### 3.2.4. Theme 4: Contextual Barriers to Integrating Caring and Technology

A total of seven nurses (10.3%) described challenges related to the contextual realities of psychiatric nursing practice. The realities of psychiatric nursing practice, including shift work, documentation requirements, and institutional routines that affect how care is provided, were mentioned in numerous comments. The nurses explained how these real-world circumstances affected their ability to interact effectively with patients and think critically about theoretical concepts. Some nurses pointed out that the wording of some questions did not accurately reflect how care was provided in team-based time-constrained psychiatric settings.

Some statements were as follows:

*“We provide nursing care continuously, not just as isolated points.”*

*“Whether or not I work overtime is unrelated to whether I understand patients.”*

These points highlighted how context shaped comprehension. Caring and technology were not understood in isolation; rather, they were mediated by workplace circumstances. Institutional structures, workloads, and time constraints frequently limited opportunities for reflection, making it challenging to connect theoretical principles to actual practice.

### 3.3. Joint Display of Quantitative and Qualitative Findings

To achieve a higher level of integration between the quantitative and qualitative strands, a joint display analysis was conducted (Table 3). The display maps specific items identified as “difficult” in the quantitative phase directly to their corresponding qualitative themes and participant quotes. By synthesizing these findings, meta-inferences were developed to explain the underlying cognitive, semantic, and contextual barriers. Following this integrated approach, we found that difficulty is not merely a matter of linguistic ambiguity but is rooted in a fundamental gap between the abstract “lexicon” of the TCCN theory and the pragmatic, often emotionally taxing, realities of psychiatric nursing practice.

## 4. Discussion

The findings of this study revealed a substantial gap: although psychiatric nurses generally endorsed the value of caring, they struggled with the abstract theoretical language used to articulate the TCCN concepts. The thematic analysis further illuminated the conceptual, linguistic, and practical sources of this interpretive difficulty.

### 4.1. Quantitative Findings: The Gap Between Value and Concept

The quantitative data indicated that psychiatric nurses registered moderate to high agreement with the TCCNI-RePract items (the median scores ranged from 5.00 to 7.00). Meanwhile, a significant subset of participants reported interpretive difficulty with these same items. Whereas high agreement scores in the presence of reported difficulty could be influenced by acquiescence bias or social desirability bias—in which participants may feel professional pressure to endorse statements reflecting nursing ideals—integrated analysis suggests a more nuanced interpretation. This discrepancy likely points to a “semantic gap” between the endorsement of professional values and the cognitive grasp of theoretical constructs. Specifically, these findings suggest that while nurses instinctively identify with the moral and professional core of “caring” (leading to high agreement), they struggle to navigate the specialized theoretical lexicon used to articulate the TCCN framework (leading to interpretive difficulty). This paradox indicates that nurses may possess “tacit knowledge” of caring rooted in clinical experience but lack the formal theoretical language to operationalize it within the specific context of technological competency. Rather than a lack of competence, this underscores a need for “translation” between abstract theory and clinical reality.

Specific items reflecting concrete and behavior-based caring actions (e.g., Q6, Q7, Q9, Q15, and Q16) were universally understood. Conversely, the most frequently cited difficult items (e.g., Q22 [caring as shared growth with patients/families, *n* = 19], Q20 [competence including the use of technology from a care perspective; *n* = 15], Q2 [caring utilizing competency with technology, *n* = 8], and Q21 [wholeness: knowing the patient as a whole person, *n* = 7]) embodied the core theoretical constructs of TCCN. This pattern aligns with Locsin’s TCCN theory, which posits that technological knowledge and care must harmonize (Q4). However, our results suggest that the relational and operational aspects of caring are grasped more easily by nurses than the ontological or epistemological articulation of how technology facilitates knowledge of the patient as a complete person [7,17,18]. The difficulty with Q22, the single most cited item, highlights a fundamental struggle in internalizing the concept of mutual growth—the existential core of TCCN—in their professional identity. This challenge may stem from the clinical education culture that historically prioritizes empirical procedure-based competence over philosophical reflection and relational knowledge [19].

### 4.2. Qualitative Findings: Sources of Interpretive Difficulty

The qualitative findings provide crucial insights into the difficulty of theoretical items identified in the quantitative analysis. The four emergent themes highlight the barriers faced by nurses in linking their practical understanding of caring to abstract TCCN concepts.

#### 4.2.1. Conceptual and Linguistic Barriers (Themes 1 and 2)

Theme 1 (“fragmented understanding of ‘technology and caring’”) explained the difficulty with items Q20, Q2, and Q4. Nurses primarily viewed “technology” as tools, equipment, or documentation systems, distinctly separate from the emotional and relational work of caring. This view directly contrasts with the TCCN paradigm, in which technology is an extension of a nurse’s competence to enhance the knowledge of viewing the patient as a whole. This conceptual fragmentation reinforces the persistent divide between the humanistic (caring) and technical (technology) aspects of nursing, suggesting that the integrative vision of TCCN has not been effectively translated into a psychiatric practice context [6].

Theme 2 (“struggles with abstract and philosophical language”) directly elucidated the difficulty with terms such as “wholeness” (Q21), “sincerity,” and “personhood.” Nurses deemed these terms ambiguous, foreign, or “too philosophical” for practical application and preferred language grounded in context and action [19,20]. This linguistic barrier acts as a filter, preventing the theoretical concepts from meaningfully entering the nurses’ professional lexicon and understanding.

#### 4.2.2. Practice and Emotional Barriers (Themes 3 and 4)

Theme 3 (“moral and emotional tensions in caring relationships”) introduced a deeper affective dimension to difficulty. Abstract statements were not processed intellectually but interpreted as moral assessments of personal competence or ethical dedication. The resultant feelings of guilt, shame, or defensiveness, which were captured in responses to Q11, indicate that caring is profoundly linked to moral identity and emotional burden of the profession. This emotional complexity suggests that understanding TCCN is not merely a cognitive task; it requires navigating ethical dilemmas, compassion fatigue, and the need to balance ideals with personal limits [10,11].

Theme 4 (“contextual barriers to integrating caring and technology”), alongside the analysis in Section 4.3, highlighted the practical constraints. Issues such as time constraints, documentation demands, and institutional routines mediated how nurses conceptualized and enacted care. The pragmatic realities of psychiatric settings, in which technology is often used for surveillance or paperwork, reinforced a functional task-oriented view of technology, making the relational view proposed by the TCCN theory feel idealistic or remote.

### 4.3. Integrated Findings: Contextual Mediation of Understanding

The combined quantitative (difficult abstract items) and qualitative (contextual themes) results indicate that organizational and environmental factors considerably mediate the perception and application of the TCCN theory. The environment requires a pragmatic approach to practice. When time is scarce and accountability is high, the functional use of technology (e.g., quick documentation) overshadows its relational potential (e.g., using data to know the whole person). Consequently, the abstract language of the TCCNI-RePract is likely to be viewed as a reflection of idealistic standards that are disconnected from day-to-day moral and practical compromises required in psychiatric care.

### 4.4. Implications for Practice and Education

The findings of this study highlight a significant semantic and contextual gap between the abstract theory of TCCN and its pragmatic application in psychiatric nursing. While this gap could be interpreted as a need for education, it also suggests a potential structural mismatch between the philosophical high-abstraction of TCCN and the acute, reality-oriented demands of psychiatric clinical settings. The current theoretical constructs, rooted in broad ontological claims, may require further cultural and clinical adaptation to resonate with the specific experiences of Japanese psychiatric nurses. In other words, interpretive difficulty should be interpreted as a call for “theoretical translation” rather than as a deficit in nursing knowledge. Consequently, there is an opportunity for targeted educational interventions aimed at bridging this divide.

Consistent with other reports [20], the observed conceptual and linguistic struggles underscore the gap in theoretically informed, caring-focused education among psychiatric nurses.

The highly cited “difficult” items (Q22, Q20, Q2, Q4, Q10, Q21, Q23, Q24) identified empirically serve as actionable anchors for curriculum development. Pedagogical approaches must bridge the cognitive–linguistic gap through (i) scaffolding concept introduction (i.e., using concrete clinically relevant vignettes to introduce abstract concepts, such as how monitoring device use can enable “knowing the whole person”), (ii) language familiarization (i.e., explicitly defining and discussing theoretical terms such as “wholeness” and “technological knowing” in the context of psychiatric patient care), and (iii) reframing technology (i.e., shifting the perception of technology from a purely technical task to an ethical and caring act that facilitates relational knowing) [21].

Incorporation of Carper’s four fundamental patterns of knowing in nursing—empirical, personal, ethical, and esthetic—provides a philosophical foundation [19]. The difficulty with the “whole person” (Q21) reflects the challenge of translating the holistic and relational aspects of care—central to Carper’s aesthetic and personal knowing—into measurable practice. The TCCN theory offers a middle-range model to operationalize these dimensions. Future research should rigorously evaluate the effectiveness of educational programs designed using evidence-based anchors [18].

### 4.5. Limitations

This study was limited to psychiatric nurses in the Kansai region of Japan, and the findings may not be generalizable to all psychiatric settings. We also acknowledge the possibility of selection bias due to the recruitment from five specific psychiatric hospitals. Furthermore, the reliance on self-reported data introduces the potential for social desirability bias and acquiescence bias. Because “caring” is a fundamental ethical pillar of the nursing profession, participants may have felt a professional obligation to provide high agreement scores on the TCCNI-RePract, regardless of their actual conceptual understanding. This likely contributed to the “paradox of agreement” observed in our integrated analysis, where high quantitative scores coexisted with qualitative reports of interpretive difficulty. While potential bias might limit the interpretability of the raw quantitative means, the mixed-methods design allowed us to use the qualitative data as a “check” on these potentially inflated scores. By identifying this discrepancy, our study moves beyond a simple evaluation of agreement to uncover the underlying “semantic gap.” Consequently, the high scores should not be viewed as evidence of theoretical mastery but rather as an endorsement of professional ideals that require further linguistic and clinical contextualization. Finally, as the analyses were exploratory and descriptive, no causal inferences can be made.

## 5. Conclusions

This concurrent mixed-methods study revealed that although psychiatric nurses strongly endorsed the core values of caring, they experienced substantial conceptual and linguistic challenges in interpreting the TCCNI-RePract. This was particularly evident in items expressing abstract theoretical constructs, such as the integration of technology with caring and the ontological view of the patient as a whole person. Conversely, concrete, behavior-oriented items were readily understood and endorsed. Thematic analysis corroborated these findings by identifying key barriers, including the fragmentation of “technology” and “caring” in clinical discourse and the presence of moral and emotional tensions that complicated the understanding of abstract concepts. These results highlight a significant semantic and contextual gap between the abstract theory of TCCN and its pragmatic application in psychiatric nursing. Rather than a simple lack of understanding, this gap suggests that theoretical constructs require more robust “translation” into the specific realities of psychiatric care. To bridge this divide, future initiatives should focus on theory-informed education—involving scaffolded or case-based approaches—that facilitates the contextualization of these abstract theoretical concepts into observable clinical practice.

## Figures and Tables

**Table 1:** Demographic characteristics of the participants (*N* = 291).

Characteristics	Median (IQR)
Age (years)	43.0 (14.0)
Years of experience as a nurse	15.0 (16.0)
Years of experience as a psychiatric nurse	12.0 (14.0)
	*N* (%)
Sex	
Men	105 (36.1)
Women	185 (63.6)
Non-binary	1 (0.3)
Final education in nursing	
Vocational school	247 (84.9)
University	21 (7.2)
High school of nursing	10 (3.4)
Junior college	10 (3.4)
Graduate school	3 (1)
Nursing qualifications	
Registered nurse	242 (83.2)
Licensed practical nurse	39 (13.4)
Certified nurse	9 (3.1)
Certified nurse specialist	1 (0.3)
Current ward	
General psychiatric ward	91 (31.3)
Psychiatric care unit	57 (19.6)
Psychiatric emergency inpatient unit (super emergency unit)	54 (18.6)
Psychiatric acute-care unit	50 (17.2)
Dementia care unit	37 (12.7)
Special disease unit	2 (0.7)
Education on caring	
No	224 (77)
Yes, I learned at a nursing training program	43 (14.8)
Yes, I learned at an outside training program	13 (4.5)
Yes, I learned at in-facility training	10 (3.4)
Other (specify)	1 (0.3)
Receiving continuous education in psychiatric nursing	
Actively receiving	7 (2.4)
Yes, I am taking it.	69 (23.7)
Can’t say either way	30 (10.3)
Not very much	76 (26.1)
No	109 (37.5)

IQR, interquartile range.

**Table 2:** Items in the perception dimension of the TCCNI-RePract that were rated as difficult to understand by participants.

#	Question	Question Items	N (%)	Median	IQR
1	Q22	Nursing as caring is the involvement of nurses with patients and their families in ways that allow them to grow together in the shared nursing situation.	19	5.00	1.75
2	Q20	Nurses’ competence includes the use of healthcare technologies from the perspective of caring in nursing.	15	6.00	1.00
3	Q2	Nurses are professionals who express caring utilizing competency with technology.	8	5.00	1.00
4	Q10	Nurses have to use technology in order to know patients as persons who are complete and to maintain honest relationships with them.	7	6.00	1.00
5	Q21	Knowing the patient is understanding the whole person.	7	6.00	2.00
6	Q25	Technology is useful for understanding patients’ health conditions.	7	6.00	1.00
7	Q3	Nurses have to provide care for patients by using necessary technologies.	6	6.00	1.00
8	Q4	Nurses must provide nursing care through the harmonious relationship between technological knowing and caring.	6	6.00	1.75
9	Q11	Nurses must finish nursing duties within a specific time even if they cannot completely know the patients, for example, their emotional needs or feelings.	6	5.00	2.00
10	Q12	Nurses must respect patients’ beliefs and focus on their recovery while anticipating their hopes, needs, and desires.	6	5.00	2.00
11	Q23	Nurses use technologies with competency in order to know patients.	6	5.00	1.00
12	Q24	Nurses use technologies with competency in order to know patients’ families.	5	5.00	2.00
13	Q1	Nurses must emphasize thoughtfulness and consideration for patients.	2	6.00	1.00
14	Q8	Nurses must share information with their patients in order to know them better.	2	6.00	2.00
15	Q13	Nurses need to maintain patients’ lifestyles and allow them to regain their healthy lives.	2	6.00	1.00
16	Q18	Nurses must consider patient’s stress and anxiety level occurring within the nurse-patient relationship.	2	6.00	1.00
17	Q19	Nurses have to know the patients, not only focusing on their physical aspects but also on accurately understanding “who they are as persons.”	2	6.00	1.00
18	Q5	Nurses need to consider providing nursing care because each patient’s wishes always change.	1	6.00	1.00
19	Q14	Nurses must emphasize thoughtful consideration of patient’s feelings, encouragement, and respect.	1	6.00	1.00
20	Q17	Nurses must act by carefully listening to the patients’ voices and expressing compassion.	1	6.00	1.00
21	Q6	Nurses must make a plan of care together with the patient to ensure quality nursing.	0	6.00	2.00
22	Q7	Nurses need to know patient’s health data in order to take care of the patient.	0	7.00	1.00
23	Q9	Nurses must provide care with a thorough understanding of their own competency.	0	6.00	1.00
24	Q15	Nurses need to provide timely nursing care in accordance with patients’ physical and emotional conditions.	0	6.00	1.00
25	Q16	Nurses must be devoted towards meeting the patient’s needs, hopes, wishes, and dreams.	0	5.00	1.00

Note: The perception dimension of the TCCNI-RePract was measured using a 7-point Likert scale, with values ranging from 1 (strongly disagree) to 7 (strongly agree). The participants completed the perception dimension of the TCCNI-RePract to assess their understanding of the instrument. IQR, interquartile range.

**Table 3:** Joint display of quantitative and qualitative findings.

Quantitative Items (Difficult to Understand)	Quantitative Metrics (n/Median)	Linked Qualitative Theme	Illustrative Participant Quote	Meta-Inference (Integration)
Q22: Nursing as caring is involvement… to grow together.	N = 19/Median = 5.00	Theme 2: Struggles with Abstract/Philosophical Language	“I was unsure how to interpret words like compassion, sincerity, hope, and dreams.”	Nurses accept the “value” of mutual growth but lack the theoretical lexicon to operationalize it in clinical settings.
Q20/Q2: Competence includes use of healthcare technologies from a caring perspective.	N = 15/Median = 6.00	Theme 1: Fragmented Understanding of “Technology and Caring”	“Technology and caring… feel like different things. Technology means physical data or infusion pumps.”	A perceptual dichotomy exists where technology is viewed as a “task-oriented tool” rather than a “medium for caring.”
Q21: Knowing the patient is understanding the whole person.	N = 7/ Median = 6.00	Theme 2: Struggles with Abstract/Philosophical Language	“It was hard to answer questions involving abstract concepts or those that could change depending on personal interpretation.”	The ontological concept of “wholeness” is perceived as subjective and impractical, leading to interpretive discomfort despite high agreement.
Q11: Nurses must finish duties… even if they cannot completely know the patient.	N = 6/ Median = 5.00	Theme 3: Moral and Emotional Tensions	“It sounded as if I was always neglecting patients… Nurses are humans too, with their own lives.”	Items intended to measure theoretical understanding instead trigger defensiveness and guilt, revealing the emotional burden of the profession.
All Theoretical Items (e.g., Q10, Q4, Q23)	High overall difficulty counts	Theme 4: Contextual Barriers to Integrating Caring and Tech	“Whether or not I work overtime is unrelated to whether I understand patients.”	Institutional constraints (time, staffing) create a pragmatic environment that reinforces task-based thinking, making TCCN theory feel “idealistic.”

## Data Availability

The data supporting the findings of this study are not publicly available because of privacy and ethical restrictions. The participants were assured that their responses would remain confidential and would be used solely for research purposes. Requests for data access may be considered by the corresponding author upon reasonable request, provided that appropriate ethical approval has been obtained.

## Supplementary Materials

The following supporting information can be downloaded at: https://www.mdpi.com/article/10.3390/nursrep16030077/s1, Table S1: STROBE Statement—Checklist of items that should be included in reports of cross-sectional studies. Table S2: Good Reporting of a Mixed Methods Study (GRAMMS) Checklist.

## Public Involvement Statement

No public involvement in any aspect of this research.

## Guidelines and Standards Statement

This manuscript was drafted against the STROBE statement for cross-sectional research and the GRAMMS checklist for mixed-methods research.

## Abbreviations

The following abbreviations are used in this manuscript:

TCCN	Technological Competency as Caring in Nursing
TCCNI-RePract	Technological Competency as Caring in Nursing Instrument–Revised for Practice
