# Supplementary material for: Exploring the Interpretive Clarity of the TCCNI-RePract and Identifying Conceptual Barriers Encountered by Japanese Psychiatric Nurses: A Concurrent Mixed-Methods Study
Source: Nurs Rep. 2026 Feb 24;16(3):77. doi: 10.3390/nursrep16030077 (PMC13029140; doi:10.3390/nursrep16030077)
Supplement: Supplementary file 1 [file nursrep-16-00077-s001.zip › nursrep-4104856-supplementary.pdf]

Table S1: STROBE Statement—Checklist of items that should be included in reports of cross-sectional studies

**Item No**	**Recommendation**	**Reported Section / Page No**
**Title and abstract**	1 (a) Indicate the study's design with a commonly used term in the title or the abstract	Title , Abstract
	(b) Provide in the abstract an informative and balanced summary of what was done and what was found	Abstract
**Introduction**		
Background/rationale	2 Explain the scientific background and rationale for the investigation being reported	1. Introduction
Objectives	3 State specific objectives, including any prespecified hypotheses	1. Introduction
**Methods**		
Study design	4 Present key elements of study design early in the paper	2.1 Research Design
Setting	5 Describe the setting, locations, and relevant dates, including periods of recruitment and data collection	2.2 Participants, Settings, and Criteria
Participants	6 (a) Give the eligibility criteria, and the sources and methods of selection of participants	2.2 Participants, Settings, and Criteria
Variables	7 Clearly define all outcomes, exposures, predictors, potential confounders, and effect modifiers	2.3 Instrumentation , 2.5 Data Analysis
Data sources/ measurement	8 For each variable of interest, give sources of data and details of methods of assessment (measurement)	2.3 Instrumentation , 2.4 Data Collection

Item No	Recommendation	Reported Section / Page No
Bias	9 Describe any efforts to address potential sources of bias	2.2 , 2.4 , 4.5 Limitations
Study size	10 Explain how the study size was arrived at	2.2
Quantitative variables	11 Explain how quantitative variables were handled in the analyses	2.5 Data Analysis
Statistical methods	12 (a) Describe all statistical methods, including those used to control for confounding	2.5 Data Analysis
**Results**		
Participants	13 (a) Report numbers of individuals at each stage of study	2.2 , 3.1.1
Descriptive data	14 (a) Give characteristics of study participants and information on exposures and potential confounders	Table 1
Outcome data	15 Report numbers of outcome events or summary measures	3.1.3 , Table 3
Main results	16 (a) Give unadjusted estimates and, if applicable, confounder-adjusted estimates and their precision	3.1.2 , Table 2
Other analyses	17 Report other analyses done—e.g., analyses of subgroups and interactions, and sensitivity analyses	3.3 Joint Display , Table 4
**Discussion**		
Key results	18 Summarise key results with reference to study objectives	5. Conclusions
Limitations	19 Discuss limitations of the study, taking into account sources of potential bias or imprecision	4.5 Limitations

Item No	Recommendation	Reported Section / Page No
Interpretation	20 Give a cautious overall interpretation of results considering objectives, limitations, and other relevant evidence	4. Discussion
Generalisability	21 Discuss the generalisability (external validity) of the study results	4.5 Limitations
**Other information**		
Funding	22 Give the source of funding and the role of the funders	Funding

Table S2: Good Reporting of A Mixed Methods Study (GRAMMS) Checklist

**No**	**Quality Guideline**	**Reported Section / Page No**
1	Describe the justification for using a mixed methods approach to the research question	2.1 , 2.5
2	Describe the design in terms of the purpose, priority and sequence of methods	2.1 , 2.5
3	Describe each method in terms of sampling, data collection and analysis	2.2 , 2.4 , 2.5
4	Describe where integration has occurred, how it has occurred and who has participated in it	2.5 , 3.3 Joint Display , Table 4
5	Describe any limitation of one method associated with the presence of the other method	4.5 Limitations
6	Describe any insights gained from mixing or integrating methods	3.3 , 4.3 , 5. Conclusions
